# Serial evaluation of SOFA and APACHE II scores to predict neurologic outcomes of out-of-hospital cardiac arrest survivors with targeted temperature management

**DOI:** 10.1371/journal.pone.0195628

**Published:** 2018-04-05

**Authors:** Jae Chol Yoon, Youn-Jung Kim, You-Jin Lee, Seung Mok Ryoo, Chang Hwan Sohn, Dong-Woo Seo, Yoon-Seon Lee, Jae Ho Lee, Kyoung Soo Lim, Won Young Kim

**Affiliations:** 1 Department of Emergency Medicine, Research Institute of Clinical Medicine of Chonbuk National University and Biomedical Research Institute of Chonbuk National University Hospital, Jeonju-si, Republic of Korea; 2 Department of Emergency Medicine, University of Ulsan College of Medicine, Asan Medical Center, Seoul, Korea; 3 Department of Emergency Medicine, University of Ulsan College of Medicine, Gangneung Asan Hospital, Gangneung, Korea; Azienda Ospedaliero Universitaria Careggi, ITALY

## Abstract

**Objective:**

This study was aimed at a serial evaluation and comparison of the prognostic values of Sequential Organ Failure Assessment (SOFA) and Acute Physiology and Chronic Health Evaluation (APACHE) II scores for neurologic outcomes in comatose, out-of-hospital cardiac arrest (OHCA) survivors, treated with targeted temperature management (TTM).

**Methods:**

We analysed a prospective cohort of comatose OHCA patients, with TTM, admitted to an emergency intensive care unit (ICU), between January 2010 and December 2015. SOFA and APACHE II scores were calculated initially, and then at day 1, 2, 3, 5, and 7 after ICU admission. Primary and secondary outcomes were the 28-day neurologic outcome and the 28-day mortality, respectively. Prognostic value of the SOFA and APACHE II scores was analysed using the receiver operating characteristic curve.

**Results:**

Of the 143 selected patients, 62 survived and 34 had good neurologic outcomes at day 28. There was no significant difference in the SOFA and extracerebral SOFA scores between the good and poor neurologic outcome groups. However, the APACHE II scores were significantly higher in the good outcome group; they displayed good discriminatory power in predicting poor outcomes, unlike the SOFA scores. The APACHE II score at day 3 had the highest prognostic value for predicting poor neurologic outcomes with an area under the cure of 0.793, and with a cut-off value of 20, the APACHE II score predicted poor neurologic outcomes with a sensitivity of 43.75%, a specificity of 94.12%, a positive predictive value of 94.59%, and a negative predictive value of 41.56%.

**Conclusions:**

Identifying APACHE II score might assist as one piece of multimodal prognostic approach for the assessment of neurologic outcomes in OHCA survivors treated with TTM.

## Introduction

Out-of-hospital cardiac arrest (OHCA) is a major public health concern, with a global average incidence of 55 adult OHCAs of a presumed cardiac cause, per 100,000 person-years [1]. In spite of the application of new and effective therapeutic interventions, as well as the fact that guidelines have been updated, the outcomes associated with OHCAs remain dismal, with only 10% of the patients surviving until hospital discharge and 5% experiencing full neurologic recovery [2, 3]. Following successful resuscitation, cardiovascular dysfunction, global ischaemia-reperfusion, and systemic inflammation contribute further to incidences of multiple organ dysfunction and brain injury [4]. This response termed ‘post-cardiac arrest syndrome’ varies by the duration and cause of cardiac arrest [5].

Although brain injury accounts for most of these deaths, whole-body ischaemia-reperfusion injury causes the activation of immunologic and coagulation pathways, leading to multiple organ failure [6]. This type of multiple organ failure may be related to patient outcomes, but there is no recommended assessment tool for the routine measurement of its severity. Recent studies reported the presence of extracerebral organ dysfunction in 66% of patients with post-cardiac arrest, and further found that the Sequential Organ Failure Assessment (SOFA) scores of the cardiovascular system were independently associated with in-hospital mortality [7, 8] (S1 Table). However, it is still not known if multiple organ dysfunction is associated with neurologic outcomes in comatose OHCA survivors treated with targeted temperature management (TTM).

Although the Acute Physiology and Chronic Health Evaluation (APACHE) II score (S2 Table), one of the most well-known illness severity scores [9], was not previously validated for use specifically in OHCA survivors treated with TTM, it has been validated as a useful instrument for predicting morbidity and mortality in critically ill patients [10, 11]. We hypothesized that APACHE II scores would be more suitable than SOFA scores in the assessment of outcomes in post-cardiac arrest patients, as they include age, comorbidities and physiological parameters. The objective of this study was to conduct a serial evaluation and comparison of the prognostic values of SOFA and APACHE II scores for neurologic outcomes, in comatose OHCA patients treated with TTM.

## Materials and methods

### Study design and patients

This single-centre retrospective, observational, registry-based study was performed at the emergency intensive care unit (ICU) of a university-affiliated teaching hospital in Korea. Data were extracted from the OHCA registry, which prospectively collected data of consecutive patients with OHCA, between January 2010 and December 2015. All OHCA survivors in our institution are entered into OHCA registry. The institutional review board of the University of Ulsan College of Medicine reviewed the study protocol and approved the study (approval number: 2016–0476). Informed consent was waived due to the retrospective nature of this study. The cohort included successfully resuscitated patients above the age of 18 years, who experienced non-traumatic OHCA with subsequent cardiopulmonary resuscitation, in whom the return of spontaneous circulation (ROSC) was achieved, who had neurologic impairments immediately after ROSC (defined as a patient’s inability to follow commands), and who were treated with therapeutic hypothermia [12, 13].

### Management and data collection

All eligible patients were admitted to the emergency ICU. Post-resuscitation care, including coronary reperfusion or TTM, in accordance with the then-current advanced cardiac life support guidelines, was provided to them. TTM was performed using Arctic Sun Energy Transfer Pads [Medivance Corp, Louisville, Colo], with the aim of achieving a body temperature of 33–36 °C. The target temperature was maintained for 24 hours and then patients were rewarmed at a rate of 0.25°C/h. During TTM, the temperature was monitored using an oesophageal temperature probe. Propofol and opioids (morphine or remifentanil) were used for sedation and analgesia. Neuromuscular blockades were administered to control shivering, if the need arose. All the patients received standard intensive care, according to the institutional ICU protocols.

Data on the following variables were obtained from the registry: age, sex, pre-existing illnesses, presence of a witness on collapse, first monitored rhythm, aetiology of cardiac arrest, collapse-to-ROSC interval, initial core temperature, pre-induction time, induction time, rewarming time, vital status, mortality (alive or dead), and Cerebral Performance Category (CPC) score at 28 days. In addition, we calculated the SOFA and APACHE II scores during the first 7 days after the ROSC. The SOFA score has a range of 0–4 points for each of the 6 organ systems (respiratory, coagulation, hepatic, cardiovascular, neurologic, and renal) [14]. The APACHE II score is composed of 12 physiological variables and 2 disease-related variables [9]. We also calculated the extracerebral SOFA score by excluding the neurologic component from the original SOFA score because the neurologic component has the potential to overshadow the other organ systems. We determined the SOFA and APACHE II scores, for each post-cardiac arrest patient, initially and then, at day 1, day 2, day 3, day 5, and day 7 after ICU admission. The initial scores were determined using the first value obtained after the ROSC; to determine the scores for days 1, 2, 3, 5, and 7, we used the worst value for each component during each 24-hour period. The primary outcome was the 28-day neurologic outcome, measured on the CPC scale. A poor neurologic outcome was defined as a CPC score of 3–5.

### Statistical analysis

Continuous and categorical variables are represented as median with interquartile range (IQR), and number (%), respectively. Comparisons between patients were performed using the Mann–Whitney *U* test for continuous variables and the Chi-square test or Fisher’s exact test for categorical variables. To estimate the effect of both day and group on the scores, we performed the linear mixed model that accounted for data clustering and dependency. After confirming the group-by-time interaction effect, the scores were compared the group effects within time points. All reported P-values are two sided, and P-value of <0.05 was considered statistically significant. The prognostic value of the SOFA and APACHE II scores, to predict poor neurologic outcomes, was analysed using the receiver operating characteristic curve with the area under the cure (AUC). The optimal cut-off value of the scores was determined using Youden’s index. Data manipulation and statistical analyses were conducted using SAS^®^ version 9.4 (SAS Institute Inc., Cary, NC) and IBM SPSS of Windows, version 21.0 (IBM Corp., Armonk, NY, USA).

## Results

During the study period, a total of 143 patients with OHCA were admitted to the emergency ICU for post-resuscitation care. Among them, 62 patients (43.4%) survived till the 28-day mark, and 34 patients (23.8%) had good neurologic outcomes at day 28. The demographic and clinical characteristics of the patients are summarized in Table 1. The median patient age was 61.0 years, and two-thirds of them were male (65.7%). While there was no significant difference between the survivors and non-survivors, in terms of the arrest cause and initial rhythm at the scene, the group with good neurologic outcomes more frequently showed arrests of cardiac origin (76.5% vs. 34.9%, P < 0.001) and shockable rhythms at the scene (50.0% vs. 17.4%, P < 0.001) than the group with poor neurologic outcomes (S3 Table). The median SOFA and APACHE II scores at the time of admission were 11.0 (8.0–13.0) and 26.0 (23.0–30.0), respectively.

**Table 1:** Baseline and cardiac arrest characteristics of the study patients according to neurologic outcome at 1 month.

	All patients (n = 143)	Good neurologic outcome (n = 34)	Poor neurologic outcome (n = 109)
**Demographics**			
**Age, years**	61.0 (48.0–72.0)	51.5 (40.0–64.8)	62.0 (50.0–73.5)
**Male**	94 (65.7%)	23 (67.6%)	71 (65.1%)
**Comorbidities**			
**Coronary artery disease**	20 (14.0%)	2 (5.9%)	18 (16.5%)
**Congestive heart failure**	11 (7.7%)	4 (11.8%)	7 (6.4%)
**Hypertension**	48 (33.6%)	6 (17.6%)	42 (38.5%)
**Diabetes mellitus**	36 (25.2%)	6 (17.6%)	30 (27.5%)
**Chronic lung disease**	18 (12.6%)	0 (0%)	18 (16.5%)
**Liver cirrhosis**	5 (3.5%)	0 (0%)	5 (4.6%)
**Chronic renal disease**	15 (10.5%)	0 (0%)	15 (13.8%)
**Arrest cause**			
**Cardiac**	64 (44.8%)	26 (76.5%)	38 (34.9%)
**Respiratory**	31 (21.7%)	3 (8.8%)	28 (25.7%)
**Others**	48 (33.6%)	5 (14.7%)	43 (39.4%)
**Initial rhythm at scene**			
**Shockable**	36 (25.2%)	17 (50.0%)	19 (17.4%)
**Non-shockable**	107 (74.8%)	17 (50.0%)	90 (82.6%)
**SOFA score, at admission**	11.0 (8.0–13.0)	10.0 (7.0–12.0)	11.0 (8.0–13.0)
**Respiratory**	3.0 (1.0–4.0)	3.0 (2.0–4.0)	2.0 (0.0–3.0)
**Cardiovascular**	4.0 (3.0–4.0)	3.0 (0.0–4.0)	4.0 (3.0–4.0)
**Renal**	1.0 (0.0–1.0)	0.5 (0.0–1.0)	1.0 (0.0–2.0)
**Coagulation**	0.0 (0.0–0.0)	0.0 (0.0–0.3)	0.0 (0.0–0.5)
**Hepatic**	0.0 (0.0–0.0)	0.0 (0.0–0.0)	0.0 (0.0–0.0)
**Neurologic**	4.0 (4.0–4.0)	4.0 (4.0–4.0)	4.0 (4.0–4.0)
**APACHE II score, at admission**	26.0 (23.0–30.0)	23.0 (20.8–27.0)	27.0 (24.0–31.0)

Values are presented as median with interquartile range or number (percent).

SOFA, Sequential Organ Failure Assessment; APACHE II, Acute Physiology and Chronic Health Evaluation II.

**Fig 1:**
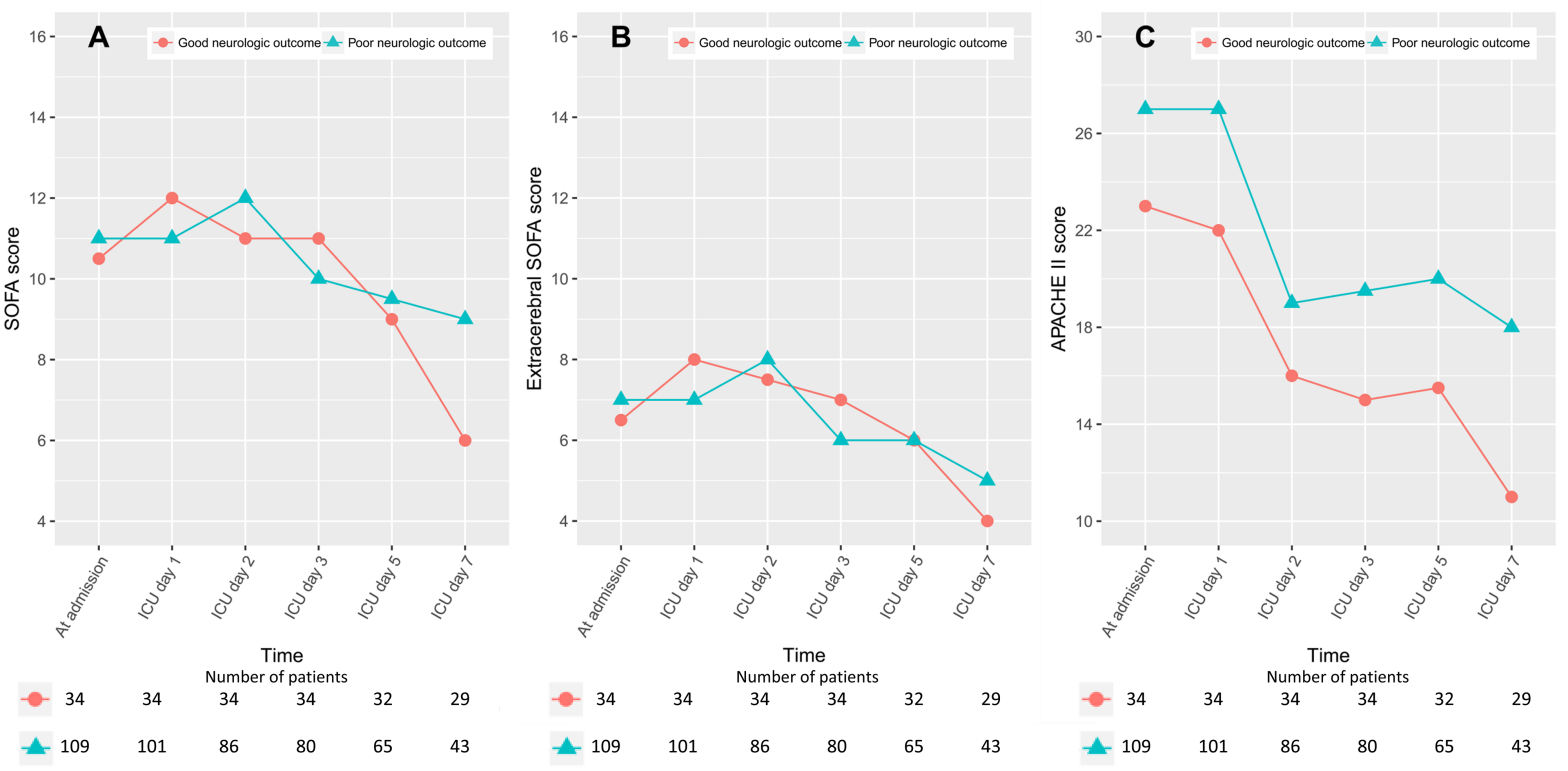
SOFA (A), extracerebral SOFA (B), and APACHE II (C) scores between the good and poor neurologic outcome groups. SOFA, Sequential Organ Failure Assessment; APACHE II, Acute Physiology and Chronic Health Evaluation II; ICU, intensive care unit.

Although the SOFA and extracerebral SOFA scores of the survivors were significantly lower than those of the non-survivors, for all the study time points, except for day 1 in the ICU (S4 Table), there was no significant difference between the good neurologic outcome and poor neurologic outcome groups, in terms of the SOFA and extracerebral SOFA scores, except for the SOFA scores at day 7 in the ICU (Fig 1A–1C). In contrast to the SOFA and extracerebral SOFA scores, a significant difference in the APACHE II scores was observed between the good neurologic outcome and the poor neurologic outcome groups, as well as between the survivors and non-survivors. The peak values of all three scoring systems were observed within the first 72 hours after admission. The linear mixed model for neurologic outcome showed that the group-by-time interaction was significant for SOFA score (P < 0.001) and not for extracerebral SOFA (P = 0.06) and APACHE II (P = 0.37) scores (S5 Table). For all time points, the least square mean of APACHE II score was also significantly lower in the good neurologic outcome group.

**Fig 2:**
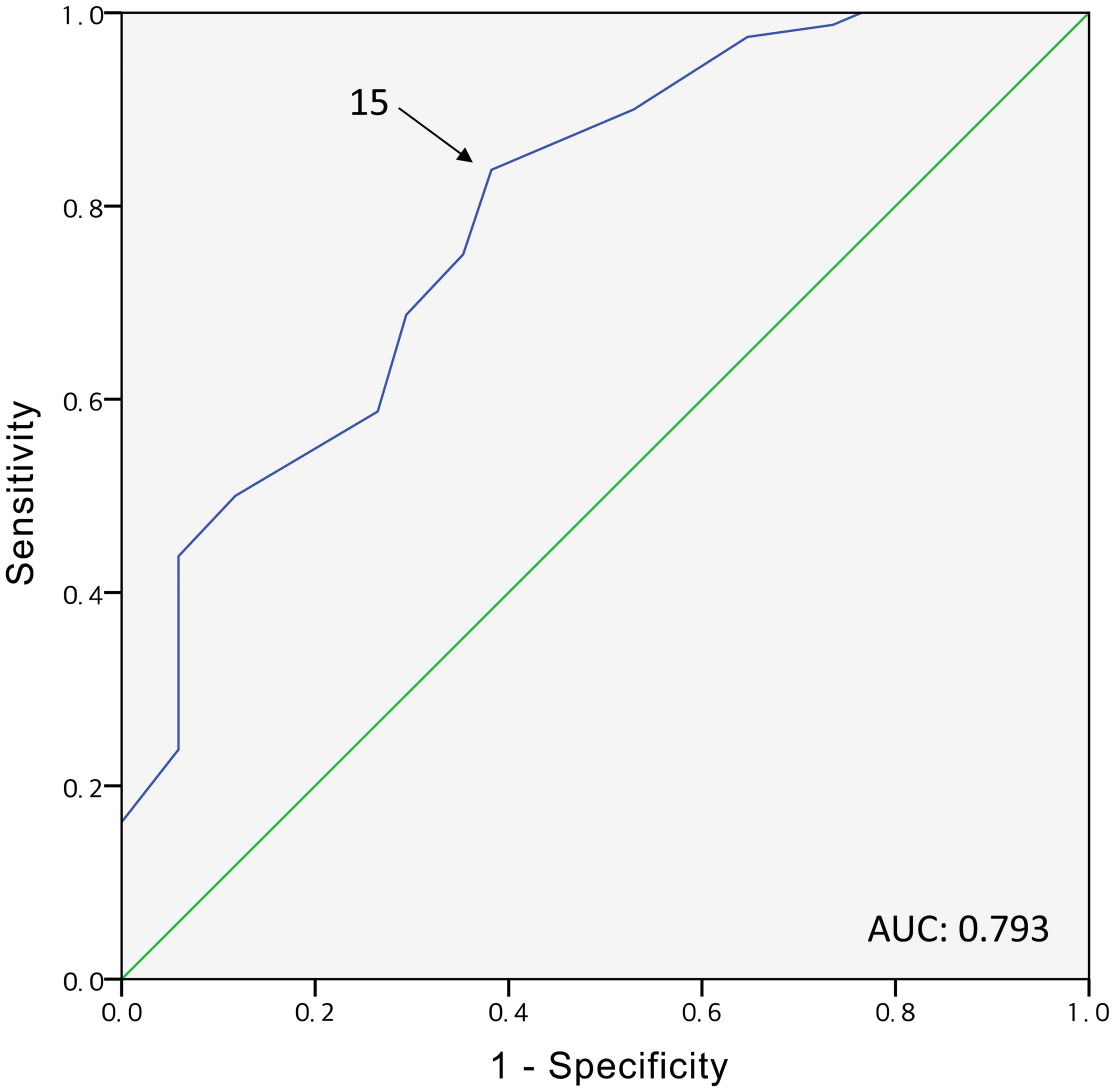
Receiver operating characteristic curve of the APACHE II score for day 3 in the intensive care unit, to predict poor neurologic outcomes. APACHE II, Acute Physiology and Chronic Health Evaluation II.

The performance of the SOFA, extracerebral SOFA, and APACHE II scores in predicting 28-day mortality and poor neurologic outcomes is presented in Table 2. While the discriminatory power, in terms of predicting 28-day mortality during the first 72 hours after admission, was weak for the SOFA scores (AUC, range: 0.563–0.644) and extracerebral SOFA scores (AUC, range: 0.567–0.619), there was no discriminatory power, in terms of the prediction of 28-day poor neurologic outcomes, for these scores. In contrast, the APACHE II scores showed a fair level of discriminatory power in predicting both 28-day mortality and poor neurologic outcomes; they showed higher AUC values in predicting poor neurologic outcomes than in predicting mortality. The power of predicting poor neurologic outcomes was highest on day 3 in the ICU (AUC, 0.793) and the optimal cut-off point was 15 (Fig 2). The predictive performance of the APACHE II scores, with different cut-off values, was evaluated for all the study time points (Table 3). With a cut-off score of 15, on day 3 in the ICU, the APACHE II score predicted 28-day poor neurologic outcomes with a sensitivity of 83.75%, a specificity of 61.76%, a positive predictive value of 83.75%, and a negative predictive value of 61.76%. The APACHE II score with cut-off value of 20 at day 3 predicted poor neurologic outcomes with a sensitivity of 43.75%, a specificity of 94.12%, a positive predictive value of 94.59%, and a negative predictive value of 41.56%.

**Table 2:** Area under the curve of the receiver operating characteristic curves of the SOFA and APACHE II scores, for predicting 28-day mortality and poor neurologic outcome.

		28-day mortality		28-day poor neurologic outcome	
	Number	AUC	95% CI	AUC	95% CI
**SOFA score**					
**At admission**	143	0.634	0.541–0.726	0.572	0.463–0.682
**ICU day 1**	135	0.563	0.467–0.660	0.427	0.327–0.527
**ICU day 2**	120	0.628	0.526–0,730	0.526	0.416–0.636
**ICU day 3**	114	0.644	0.543–0.745	0.485	0.373–0.598
**ICU day 5**	98	0.680	0.571–0.788	0.529	0.409–0.648
**ICU day 7**	73	0.731	0.586–0.841	0.642	0.503–0.781
**Extracerebral SOFA score**					
**At admission**	143	0.619	0.525–0.713	0.557	0.445–0.668
**ICU day 1**	135	0.567	0.471–0.664	0.420	0.320–0.521
**ICU day 2**	120	0.607	0.505–0.710	0.482	0.372–0.592
**ICU day 3**	114	0.618	0.514–0.721	0.431	0.324–0.537
**ICU day 5**	98	0.652	0.540–0.763	0.478	0.360–0.596
**ICU day 7**	73	0.674	0.536–0.812	0.559	0.419–0.698
**APACHE II score**					
**At admission**	143	0.637	0.546–0.729	0.697	0.599–0.795
**ICU day 1**	135	0.677	0.587–0.766	0.716	0.618–0.813
**ICU day 2**	120	0.665	0.569–0.761	0.695	0.590–0.800
**ICU day 3**	114	0.694	0.599–0.789	0.793	0.702–0.884
**ICU day 5**	98	0.750	0.654–0.846	0.761	0.654–0.867
**ICU day 7**	73	0.719	0.599–0.839	0.786	0.669–0.903

AUC, area under the curve; CI, confidence interval; SOFA, Sequential Organ Failure Assessment; APACHE II, Acute Physiology and Chronic Health Evaluation II; ICU, Intensive Care Unit.

**Table 3:** Sensitivity, specificity, and positive and negative predictive values at different cut-off values of the APACHE II score, for predicting mortality and poor neurologic outcome.

APACHE II Cut-off value	AUC	Sensitivity	Specificity	Positive Predictive Value	Negative Predictive Value	Positive Likelihood Ratio	Negative Likelihood Ratio
**28-day mortality**							
**At admission**							
**> 24**	0.637	78.90%	52.94%	84.31%	43.90%	1.68	0.40
**ICU day 1**							
**> 25**	0.680	56.44%	79.41%	89.06%	38.03%	2.74	0.55
**ICU day 2**							
**> 15**	0.665	87.93%	32.26%	54.84%	74.07%	1.30	0.37
**ICU day 3**							
**> 16**	0.694	75.00%	64.71%	83.33%	52.38%	2.13	0.39
**ICU day 5**							
**> 19**	0.750	55.38%	84.38%	87.80%	48.21%	3.55	0.53
**ICU day 7**							
**> 14**	0.719	79.07%	68.97%	79.07%	68.97%	2.55	0.30
**28-day poor neurologic outcome**							
**At admission**							
**> 23**	0.697	78.90%	52.94%	84.31%	43.90%	1.68	0.40
**ICU day 1**							
**> 25**	0.719	56.44%	79.41%	89.06%	38.03%	2.74	0.55
**ICU day 2**							
**> 21**	0.695	39.53%	88.24%	89.47%	36.59%	3.36	0.69
**ICU day 3**							
**> 15**	0.793	83.75%	61.76%	83.75%	61.76%	2.19	0.26
**> 20**	0.793	43.75%	94.12%	94.59%	41.56%	7.44	0.60
**ICU day 5**							
**> 17**	0.761	72.31%	71.88%	83.93%	56.10%	2.57	0.39
**ICU day 7**							
**> 12**	0.786	90.70%	58.62%	76.47%	80.95%	2.19	0.16

APACHE II, Acute Physiology and Chronic Health Evaluation II; ICU, Intensive Care Unit.

## Discussion

In this study, we conducted the serial evaluation of SOFA and APACHE II scores in comatose OHCA patients treated with TTM and evaluated the predictive power of both scoring systems for 28-day mortality and poor neurologic outcomes. Despite significant differences in the SOFA scores between survivors and non-survivors, this score was found to be an ineffective tool for the discrimination of poor neurologic outcomes. Compared with the SOFA score, the APACHE II score was found to be a good predictor of poor neurologic outcomes as well as mortality; the performance of APACHE II scores in predicting poor neurologic outcomes was the best on day 3 in the ICU, with an AUC of 0.793. Although a substantial number of patients (29/143, 20.3%) were excluded from the analysis due to death on day 3 in the ICU, APACHE II scores, at a cut-off value of 20, predicted poor neurologic outcomes with a sensitivity of 43.75%, a specificity of 94.12%, a positive predictive value of 94.59%, and a negative predictive value of 41.56%.

The SOFA scores displayed an acceptable discriminatory power for survivors, at admission, with an AUC value of 0.634, which was consistent with that of previously conducted studies [8, 15, 16]. Respiratory, cardiovascular and neurological dysfunction were the most commonly observed types of organ dysfunction, as observed in our study patients; this is also similar to the results of previously conducted studies. The SOFA score was originally developed to assess the degree of organ dysfunction and severity in patients with sepsis [14]. Considering the similarities in the clinical and physiologic aspects between post-cardiac arrest patients and patients with sepsis, it is reasonable to assume that the SOFA score is a good assessment tool for cases of post-cardiac arrest. However, the SOFA score failed to discriminate the neurologic outcomes from the time of admission to day 5 in the ICU, and the late improvement of the AUC value at day 7 in the ICU may have led to biased results due to patients dropping out of the study. The Glasgow Coma Score is used to assess neurologic dysfunction in the SOFA score, but accurate neurologic evaluation is not commonly performed due to the use of sedative drugs and neuromuscular blockers in post-cardiac arrest patients treated with TTM, in the early phase. We also found that extracerebral SOFA scores showed a weaker predictive power than SOFA scores, across time points. These results imply that organ dysfunction, relating to the respiratory, coagulation, hepatic, cardiovascular, and renal systems, could be an indicator of mortality, but not of neurologic outcomes.

The APACHE II scores displayed a good predictive power for poor neurologic outcomes, across all time points and demonstrated the highest predictive value (AUC, 0.793) on day 3 in the ICU. The superiority of the APACHE II score when compared to the SOFA score could be attributed mainly to the measurements of the patients’ previous health status (chronic health problems) and demographic status (age) [17]. Only one study, till date, has assessed APACHE II scores in cases of OHCA [18]. This study revealed that APACHE II scores were poor predictors of outcomes at time zero in the case of OHCA. For cases of in-hospital cardiac arrest, APACHE II scores were a modest indicator of illness severity [18]. Our study differed from this previously-conducted study [18] in that we included all non-traumatic OHCA patients treated with TTM, making for a homogenous study cohort with standardized post-cardiac arrest management, assessed APACHE II scores until day 7, determined the 28-day outcomes, and compared the prognostic values using SOFA scores.

There are several limitations in our study. First, this was a single-center retrospective study based on a prospective cardiac arrest registry and, therefore, limitations pertaining to data gathering and analysis are inevitable. Second, despite following the standardized treatment protocol for post cardiac arrest, including the performance of TTM, the results cannot be generalized. Third, the results might be biased with regard to mortality and consequently, drop-outs in the early phase. However, we also performed the linear mixed model to estimate the effect of both day and group on the scores and compared the group effects within time points to minimize the confounding.

## Conclusions

Neurologic prognostication is an important clinical issue in the management of post-cardiac arrest patients, and is still under investigation. In this study, we found that APACHE II score after day 3 showed acceptable levels of discriminatory power, in terms of discriminating the good neurologic outcome group from the poor neurologic outcome group, as well as survivors from non-survivors. APACHE II scores calculated on day 3 in the ICU might be used as one piece of multimodal prognostic approach for predicting neurologic outcomes in post cardiac arrest patients treated with TTM following an OHCA. Further multicenter studies would be warranted to validate our results.

## Supporting information

S1 Table Sequential Organ Failure Assessment (SOFA) score. ^a^Catecholamine doses are given as μg/kg/min for at least 1 hour. PaO_2_, partial pressure of oxygen; FiO_2_, fraction of inspired oxygen; MAP, mean arterial pressure.
(DOCX)

S2 Table Acute Physiology and Chronic Health Evaluation (APACHE) II score. MAP, mean arterial pressure; A-aDO_2_, alveolar-arterial oxygen gradient; FiO_2_, fraction of inspired oxygen; PaO_2_, partial pressure of oxygen; ABGs, arterial blood gases.
(DOCX)

S3 Table Baseline and cardiac arrest characteristics of the study patients according to survival at 1 month. Values are presented as median with interquartile range or number (percent). SOFA, Sequential Organ Failure Assessment; APACHE II, Acute Physiology and Chronic Health Evaluation II.
(DOCX)

S4 Table Comparison of SOFA, extracerebral SOFA, and APACHE II scores in the study patients according to survival and the neurologic outcome at 1 month. Values are presented as median with interquartile range. SOFA, Sequential Organ Failure Assessment; APACHE II, Acute Physiology and Chronic Health Evaluation II; ICU, Intensive Care Unit.
(DOCX)

S5 Table Linear mixed model of SOFA, extracerebral SOFA, and APACHE II scores in the study patients according to the neurologic outcome at 1 month. SOFA, Sequential Organ Failure Assessment; APACHE II, Acute Physiology and Chronic Health Evaluation II; ICU, Intensive Care Unit; SE, standard error; CI, confidence interval.
(DOCX)
