# Supplementary material for: Serial evaluation of SOFA and APACHE II scores to predict neurologic outcomes of out-of-hospital cardiac arrest survivors with targeted temperature management
Source: PLoS One. 2018 Apr 5;13(4):e0195628. doi: 10.1371/journal.pone.0195628 (PMC5886591; doi:10.1371/journal.pone.0195628)
Supplement: S1 Table — aCatecholamine doses are given as μg/kg/min for at least 1 hour. PaO2, partial pressure of oxygen; FiO2, fraction of inspired oxygen; MAP, mean arterial pressure. (DOCX) [file pone.0195628.s001.docx]

**Supplement Table 1.** Sequential Organ Failure Assessment (SOFA) score.

|  | **Score** |  |  |  |  |
| --- | --- | --- | --- | --- | --- |
| **System** | **0** | **1** | **2** | **3** | **4** |
| **Respiration** |  |  |  |  |  |
| PaO_2_/FiO_2_, mmHg | ≥400 | <400 | <300 | <200 with respiratory support | <100 with respiratory support |
| **Coagulation** |  |  |  |  |  |
| Platelets, $\times$10^3^/µL | ≥150 | <150 | <100 | <50 | <20 |
| **Liver** |  |  |  |  |  |
| Bilirubin, mg/dL | <1.2 | 1.2-1.9 | 2.0-5.9 | 6.0-11.9 | >12.0 |
| **Cardiovascular^a^** | MAP ≥70 mmHg | MAP <70 mmHg | Dopamine <5 or dobutamine (any dose) | Dopamine 5.1-15 or epinephrine ≤0.1 or norepinephrine ≤0.1 | Dopamine >15 or epinephrine >0.1 or norepinephrine >0.1 |
| **Central nervous system** |  |  |  |  |  |
| Glasgow Coma Scale | 15 | 13-14 | 10-12 | 6-9 | <6 |
| **Renal** |  |  |  |  |  |
| Creatinine, mg/dL | <1.2 | 1.2-1.9 | 2.0-3.4 | 3.5-4.9 | >5.0 |
| Urine output, mL/d |  |  |  | <500 | <200 |

^a^ Catecholamine doses are given as µg/kg/min for at least 1 hour.

PaO_2_, partial pressure of oxygen; FiO_2_, fraction of inspired oxygen; MAP, mean arterial pressure
