# Supplementary material for: Serial evaluation of SOFA and APACHE II scores to predict neurologic outcomes of out-of-hospital cardiac arrest survivors with targeted temperature management
Source: PLoS One. 2018 Apr 5;13(4):e0195628. doi: 10.1371/journal.pone.0195628 (PMC5886591; doi:10.1371/journal.pone.0195628)
Supplement: S2 Table — MAP, mean arterial pressure; A-aDO2, alveolar-arterial oxygen gradient; FiO2, fraction of inspired oxygen; PaO2, partial pressure of oxygen; ABGs, arterial blood gases. (DOCX) [file pone.0195628.s002.docx]

**Supplement Table 2.** Acute Physiology and Chronic Health Evaluation (APACHE) II score.

|  | **Score** |  |  |  |  |
| --- | --- | --- | --- | --- | --- |
| **Physiologic Variable** | **0** | **1** | **2** | **3** | **4** |
| Temperature (rectal), °C | 36.0-38.4 | 34.0-35.9 or 38.5-38.9 | 32.0-33.9 | 30.0-31.9 or 39.0-40.9 | ≤29.9 or ≥41.0 |
| MAP (mmHg) | 70-109 |  | 50-69 or 110-129 | 130-159 | ≤49 or ≥160 |
| Heart rate (/minute) | 70-109 |  | 55-69 or 110-139 | 40-54 or 140-179 | ≤39 or ≥180 |
| Respiratory rate (/minute) | 12-24 | 10-11 or 25-34 | 6-9 | 35-49 | ≤5 or ≥50 |
| Oxygenation |  |  |  |  |  |
| A-aDO_2_ (FiO2 >0.5) | <200 |  | 200-349 | 350-499 | ≥500 |
| PaO_2_ (FiO2 ≤0.5) | >70 | 61-70 |  | 55-60 | <55 |
| Arterial pH | 7.33-7.49 | 7.50-7.59 | 7.25-7.32 | 7.15-7.24 or 7.60-7.69 | <7.15 or ≥7.70 |
| Serum sodium (mmol/L) | 130-149 | 150-154 | 120-129 or 155-159 | 111-119 or 160-179 | ≤110 or ≥180 |
| Serum potassium (mmol/L) | 3.5-5.4 | 3.0-3.4 or 5.5-5.9 | 2.5-2.9 | 6.0-6.9 | <2.5 or ≥7.0 |
| Serum creatinine (mg/dL)  (Double point score for acute renal failure) | 0.6-1.4 |  | <0.6 or 1.5-1.9 | 2.0-3.4 | ≥3.5 |
| Hematocrit (%) | 30.0-45.9 | 46.0-49.9 | 20.0-29.9 or 50.0-59.9 |  | <20.0 or ≥60.0 |
| White blood count ($\times$10^3^/mm^3^) | 3.0-14.9 | 15.0-19.9 | 1.0-2.9 or 20.0-39.9 |  | <1.0 or ≥40.0 |
| Glasgow Coma Scale | Score = 15 minus actual Glasgow Coma Scale | | | | |
| Serum HCO_3_ (venous, mmol/L, use if no ABG) | 22.0-31.9 | 32.0-40.9 | 18.0-21.9 | 15.0-17.9 or 41.0-51.9 | <15.0 or ≥52.0 |
| **A= Total Acute Physiology Score (APS)** | Sum of the 12 individual variable points. | | | | |
| **B = Age Points**  ≤44 years 0 points  45-54 years 2 points  55-64 years 3 points  65-74 years 5 points  ≥75 years 6 points | **C = Chronic Health Points**  1) Cirrhosis of the liver confirmed by biopsy; 2) New York Heart Association Class IV; 3) Severe chronic obstructive pulmonary disease-- Hypercapnia, home O2 use, or pulmonary hypertension; 4) On regular dialysis; 5) Immunocompromised | | | | |
|  | Non-surgical 5 points  Emergent operation 5 points  Elective operation 2 points | | | | |
| **APACHE II score = Sum of A (APS points) + B (Age points) + C (Chronic Health points)** | | | | | |

MAP, mean arterial pressure; A-aDO_2_, alveolar-arterial oxygen gradient; FiO_2_, fraction of inspired oxygen; PaO_2_, partial pressure of oxygen; ABGs, arterial blood gases.
