# Supplementary material for: Serial evaluation of SOFA and APACHE II scores to predict neurologic outcomes of out-of-hospital cardiac arrest survivors with targeted temperature management
Source: PLoS One. 2018 Apr 5;13(4):e0195628. doi: 10.1371/journal.pone.0195628 (PMC5886591; doi:10.1371/journal.pone.0195628)
Supplement: S3 Table — Values are presented as median with interquartile range or number (percent). SOFA, Sequential Organ Failure Assessment; APACHE II, Acute Physiology and Chronic Health Evaluation II. (DOCX) [file pone.0195628.s003.docx]

**Supplement Table 3.** Baseline and cardiac arrest characteristics of the study patients according to survival at 1 month.

|  | **All patients**  **(n=143)** | **Survivors**  **(n=62)** | **Non-survivors**  **(n=81)** |
| --- | --- | --- | --- |
| Demographics |  |  |  |
| Age, years | 61.0 (48.0-72.0) | 56.5 (41.0-68.5) | 64.0 (50.5-73.5) |
| Male | 94 (65.7%) | 40 (64.5%) | 54 (66.7%) |
| Comorbidities |  |  |  |
| Coronary artery disease | 20 (14.0%) | 4 (6.5%) | 16 (19.8%) |
| Congestive heart failure | 11 (7.7%) | 7 (11.3%) | 4 (4.9%) |
| Hypertension | 48 (33.6%) | 17 (27.4%) | 31 (38.3%) |
| Diabetes mellitus | 36 (25.2%) | 15 (24.2%) | 21 (25.9%) |
| Chronic lung disease | 18 (12.6%) | 6 (9.7%) | 12 (14.8%) |
| Liver cirrhosis | 5 (3.5%) | 1 (1.6%) | 4 (4.9%) |
| Chronic renal disease | 15 (10.5%) | 2 (3.2%) | 13 (16.0%) |
| Arrest cause |  |  |  |
| Cardiac | 64 (44.8%) | 34 (54.8%) | 30 (37.0%) |
| Respiratory | 31 (21.7%) | 10 (16.1%) | 21 (25.9%) |
| Others | 48 (33.6%) | 18 (29.0%) | 30 (37.0%) |
| Initial rhythm at scene |  |  |  |
| Shockable | 36 (25.2%) | 20 (32.3%) | 16 (19.8%) |
| Non-shockable | 107 (74.8%) | 42 (67.7%) | 65 (80.2%) |
| SOFA score, at admission | 11.0 (8.0-13.0) | 9.5 (7.0-12.0) | 12.0 (9.0-13.0) |
| Respiratory | 3.0 (1.0-4.0) | 3.0 (1.0-4.0) | 2.0 (1.0-3.0) |
| Cardiovascular | 4.0 (3.0-4.0) | 3.0 (0.0-4.0) | 4.0 (3.0-4.0) |
| Renal | 1.0 (0.0-1.0) | 1.0 (0.0-1.0) | 1.0 (0.0-2.0) |
| Coagulation | 0.0 (0.0-0.0) | 0.0 (0.0-0.0) | 0.0 (0.0-1.0) |
| Hepatic | 0.0 (0.0-0.0) | 0.0 (0.0-0.0) | 0.0 (0.0-0.0) |
| Neurologic | 4.0 (4.0-4.0) | 4.0 (4.0-4.0) | 4.0 (4.0-4.0) |
| APACHE II score, at admission | 26.0 (23.0-30.0) | 24.5 (21.0-29.0) | 28.0 (24.0-31.0) |

Values are presented as median with interquartile range or number (percent).

SOFA, Sequential Organ Failure Assessment; APACHE II, Acute Physiology and Chronic Health Evaluation II.
