# Supplementary material for: Serial evaluation of SOFA and APACHE II scores to predict neurologic outcomes of out-of-hospital cardiac arrest survivors with targeted temperature management
Source: PLoS One. 2018 Apr 5;13(4):e0195628. doi: 10.1371/journal.pone.0195628 (PMC5886591; doi:10.1371/journal.pone.0195628)
Supplement: S4 Table — Values are presented as median with interquartile range. SOFA, Sequential Organ Failure Assessment; APACHE II, Acute Physiology and Chronic Health Evaluation II; ICU, Intensive Care Unit. (DOCX) [file pone.0195628.s004.docx]

**Supplement Table 4.** Comparison of SOFA, extracerebral SOFA, and APACHE II scores in the study patients according to survival and the neurologic outcome at 1 month

|  | **Survivors** | **Non-survivors** | **P-value** | **Good neurologic outcome** | **Poor neurologic outcome** | **P-value** |
| --- | --- | --- | --- | --- | --- | --- |
| SOFA score |  |  |  |  |  |  |
| At admission | 9.5 (7.0-12.0) n=62 | 12.0 (9.0-13.0), n=81 | 0.006 | 10.5 (7.0-12.0) n=34 | 11.0 (8.0-13.0), n=109 | 0.201 |
| ICU day 1 | 11.0 (9.0-13.0) n=62 | 12.0 (9.0-15.0), n=73 | 0.204 | 12.0 (11.0-15.0) n=34 | 11.0 (8.5-14.0), n=101 | 0.163 |
| ICU day 2 | 11.0 (9.0-13.0) n=62 | 13.0 (9.8-15.0), n=58 | 0.015 | 11.0 (9.8-13.0) n=34 | 12.0 (9.0-14.0), n=86 | 0.657 |
| ICU day 3 | 9.5 (7.0-11.3) n=62 | 11.0 (8.3-14.8), n=52 | 0.008 | 11.0 (8.0-13.0) n=34 | 10.0 (8.0-13.8), n=80 | 0.804 |
| ICU day 5 | 8.0 (6.0-12.0) n=54 | 12.0 (8.0-15.0), n=43 | 0.002 | 9.0 (7.0-13.5) n=32 | 9.5 (7.0-15.0), n=65 | 0.647 |
| ICU day 7 | 7.0 (5.0-12.0) n=50 | 11.5 (7.0-15.3), n=22 | 0.004 | 6.0 (2.5-12.0) n=29 | 9.0 (6.0-14.0), n=43 | 0.042 |
| Extracerebral SOFA score | | | | | | |
| At admission | 5.5 (3.0-8.3) n=62 | 8.0 (5.0-9.0), n=81 | 0.014 | 6.5 (4.0-8.3) n=34 | 7.0 (4.0-9.0), n=109 | 0.318 |
| ICU day 1 | 7.0 (5.0-9.0) n=62 | 8.0 (5.0-11.0), n=73 | 0.178 | 8.0 (7.0-11.0) n=34 | 7.0 (4.5-10.0), n=101 | 0.170 |
| ICU day 2 | 7.0 (5.0-10.0) n=62 | 9.0 (6.0-11.0), n=58 | 0.042 | 7.5 (6.0-10.0) n=34 | 8.0 (5.0-10.0), n=86 | 0.757 |
| ICU day 3 | 6.0 (4.0-8.0) n=62 | 7.0 (5.0-10.8), n=52 | 0.030 | 7.0 (4.8-9.3) n=34 | 6.0 (4.0-9.8), n=80 | 0.240 |
| ICU day 5 | 5.0 (3.0-8.3) n=54 | 8.0 (4.0-11.0), n=43 | 0.010 | 6.0 (4.0-10.0) n=32 | 6.0 (3.0-11.0), n=65 | 0.729 |
| ICU day 7 | 4.0 (2.0-8.0) n=50 | 7.5 (3.0-11.3), n=22 | 0.019 | 4.0 (1.5-9.0) n=29 | 5.0 (3.0-10.0), n=43 | 0.400 |
| APACHE II score | | | | | | |
| At admission | 24.5 (21.0-29.0) n=62 | 28.0 (24.0-31.0), n=81 | 0.005 | 23.0 (20.8-27.0) n=34 | 27.0 (24.0-31.0), n=109 | 0.001 |
| ICU day 1 | 23.5 (20.0-27.0) n=62 | 27.0 (22.0-32.0), n=73 | <0.001 | 22.0 (18.8-25.0) n=34 | 27.0 (22.0-31.0), n=101 | <0.001 |
| ICU day 2 | 17.0 (14.0-21.0) n=62 | 19.0 (16.0-25.0), n=58 | 0.002 | 16.0 (12.0-20.0) n=34 | 19.0 (16.0-24.0), n=86 | 0.001 |
| ICU day 3 | 17.0 (14.0-20.3) n=62 | 19.5 (17.0-23.0), n=52 | <0.001 | 15.0 (11.8-19.0) n=34 | 19.5 (16.3-22.0), n=80 | <0.001 |
| ICU day 5 | 16.0 (12.8-20.0) n=54 | 21.0 (18.0-27.0), n=43 | <0.001 | 15.5 (9.0-18.8) n=32 | 20.0 (16.5-24.0), n=65 | <0.001 |
| ICU day 7 | 14.0 (7.8-19.3) n=50 | 19.0 (15.0-25.5), n=22 | 0.003 | 11.0 (6.5-15.5) n=29 | 18.0 (15.0-23.0), n=43 | <0.001 |

Values are presented as median with interquartile range.

SOFA, Sequential Organ Failure Assessment; APACHE II, Acute Physiology and Chronic Health Evaluation II; ICU, Intensive Care Unit.
