# Supplementary material for: Serial evaluation of SOFA and APACHE II scores to predict neurologic outcomes of out-of-hospital cardiac arrest survivors with targeted temperature management
Source: PLoS One. 2018 Apr 5;13(4):e0195628. doi: 10.1371/journal.pone.0195628 (PMC5886591; doi:10.1371/journal.pone.0195628)
Supplement: S5 Table — SOFA, Sequential Organ Failure Assessment; APACHE II, Acute Physiology and Chronic Health Evaluation II; ICU, Intensive Care Unit; SE, standard error; CI, confidence interval. (DOCX) [file pone.0195628.s005.docx]

**Supplement Table 5.** Linear mixed model of SOFA, extracerebral SOFA, and APACHE II scores in the study patients according to the neurologic outcome at 1 month.

| **Score** | **Group-by-day interaction  P-value** | **ICU day** | **Neurologic outcome**  **group** | **Least Square Means** | **SE** | **Estimate** | **95% CI** | | **P value** |
| --- | --- | --- | --- | --- | --- | --- | --- | --- | --- |
| SOFA | <0.001 |  |  |  |  |  |  |  |  |
|  |  | At admission | Good | 10.32 | 0.56 | 0 |  | |  |
|  |  |  | Poor | 10.84 | 0.31 | 0.51 | -0.759-1.782 | | 0.43 |
|  |  | 1 | Good | 12.35 | 0.65 | 0 |  | |  |
|  |  |  | Poor | 11.74 | 0.37 | -0.61 | -2.087-0.869 | | 0.42 |
|  |  | 2 | Good | 11.56 | 0.60 | 0 |  | |  |
|  |  |  | Poor | 12.22 | 0.36 | 0.66 | -0.721-2.041 | | 0.35 |
|  |  | 3 | Good | 10.71 | 0.70 | 0 |  | |  |
|  |  |  | Poor | 11.50 | 0.43 | 0.79 | -0.831-2.417 | | 0.34 |
|  |  | 5 | Good | 9.63 | 0.78 | 0 |  | |  |
|  |  |  | Poor | 11.08 | 0.51 | 1.45 | -0.407-3.299 | | 0.13 |
|  |  | 7 | Good | 7.10 | 0.85 | 0 |  | |  |
|  |  |  | Poor | 10.52 | 0.58 | 3.42 | 1.375-5.467 | | 0.001 |
| Extracerebral SOFA | 0.06 |  |  |  |  |  |  |  |  |
|  |  | At admission | Good | 6.53 | 0.56 | 0 |  | |  |
|  |  |  | Poor | 6.88 | 0.31 | 0.35 | -0.917-1.620 | | 0.59 |
|  |  | 1 | Good | 8.47 | 0.65 | 0 |  | |  |
|  |  |  | Poor | 7.78 | 0.37 | -0.69 | -2.162-0.784 | | 0.36 |
|  |  | 2 | Good | 8.09 | 0.59 | 0 |  | |  |
|  |  |  | Poor | 8.29 | 0.35 | 0.20 | -1.156-1.555 | | 0.77 |
|  |  | 3 | Good | 7.41 | 0.68 | 0 |  | |  |
|  |  |  | Poor | 7.56 | 0.41 | 0.15 | -1.422-1.719 | | 0.85 |
|  |  | 5 | Good | 6.73 | 0.74 | 0 |  | |  |
|  |  |  | Poor | 7.25 | 0.48 | 0.53 | -1.218-2.270 | | 0.55 |
|  |  | 7 | Good | 5.00 | 0.76 | 0 |  | |  |
|  |  |  | Poor | 6.69 | 0.51 | 1.68 | -0.135-3.497 | | 0.07 |
| APACHE II | 0.37 |  |  |  |  |  |  |  |  |
|  |  | 0 | Good | 23.71 | 0.99 | 0 |  | |  |
|  |  |  | Poor | 27.62 | 0.56 | 3.92 | 1.668-6.168 | | 0.001 |
|  |  | 1 | Good | 22.06 | 1.03 | 0 |  | |  |
|  |  |  | Poor | 27.00 | 0.59 | 4.95 | 2.593-7.297 | | <0.001 |
|  |  | 2 | Good | 16.18 | 1.02 | 0 |  | |  |
|  |  |  | Poor | 21.19 | 0.62 | 5.01 | 2.642-7.374 | | <0.001 |
|  |  | 3 | Good | 14.35 | 0.90 | 0 |  | |  |
|  |  |  | Poor | 20.85 | 0.57 | 6.50 | 4.393-8.606 | | <0.001 |
|  |  | 5 | Good | 15.03 | 1.66 | 0 |  | |  |
|  |  |  | Poor | 22.58 | 1.16 | 7.55 | 3.534-11.565 | | <0.001 |
|  |  | 7 | Good | 13.35 | 2.11 | 0 |  | |  |
|  |  |  | Poor | 23.39 | 1.61 | 10.05 | 4.754-15.336 | | <0.001 |

SOFA, Sequential Organ Failure Assessment; APACHE II, Acute Physiology and Chronic Health Evaluation II; ICU, Intensive Care Unit; SE, standard error; CI, confidence interval.
